# Controlled Cortical Impact and Craniotomy Induce Strikingly Similar Profiles of Inflammatory Gene Expression, but with Distinct Kinetics

**DOI:** 10.3389/fneur.2012.00155

**Published:** 2012-10-31

**Authors:** Mouna Lagraoui, Joseph R. Latoche, Natalia G. Cartwright, Gauthaman Sukumar, Clifton L. Dalgard, Brian C. Schaefer

**Affiliations:** ^1^Department of Microbiology and Immunology, Uniformed Services UniversityBethesda, MD, USA; ^2^Center for Neuroscience and Regenerative Medicine, Uniformed Services UniversityBethesda, MD, USA; ^3^Department of Anatomy, Physiology, and Genetics, Uniformed Services UniversityBethesda, MD, USA

**Keywords:** traumatic brain injury, inflammation, genomics, glia, response to injury, mouse models, cytokines, diagnostics

## Abstract

An immediate consequence of traumatic brain injury (TBI) is the induction of an inflammatory response. Mounting data suggest that inflammation is a major contributor to TBI-induced brain damage. However, much remains unknown regarding the induction and regulation of the inflammatory response to TBI. In this study we compared the TBI-induced inflammatory response to severe parenchymal injury (controlled cortical impact) vs. mild brain injury (craniotomy) over a 21-day period. Our data show that both severe and mild brain injury induce a qualitatively similar inflammatory response, involving highly overlapping sets of effector molecules. However, kinetic analysis revealed that the inflammatory response to mild brain injury is of much shorter duration than the response to severe TBI. Specifically, the inflammatory response to severe brain injury persists for at least 21 days, whereas the response to mild brain injury returns to near baseline values within 10 days post-injury. Our data therefore imply that the development of accurate diagnostic tests of TBI severity that are based on imaging or biomarker analysis of the inflammatory response may require repeated measures over at least a 10-day period, post-injury.

## Introduction

A major component of the biological response to traumatic brain injury (TBI) is induction of inflammation. TBI, like other forms of tissue injury, induces an immune response which is a form of sterile inflammation. This type of immune response is driven by the release of intracellular antigens which are normally hidden from leukocytes (Medzhitov, 2008; Chen and Nunez, 2010). A subset of these hidden antigens is highly immunogenic, driving immune activation by many of the same mechanisms employed in responses to pathogenic organisms. Specifically, certain autologous hidden antigens bind to and activate the same pattern recognition receptors [PRRs; e.g., toll-like receptors (TLRs)] that are the receptors for specific pathogen products. For example, TLR4 serves as the receptor for both bacterial lipopolysaccharide (LPS) and a number of hidden self-antigens, including HMGB1, hyaluronan, and specific proteins of the S100 family (Medzhitov, 2008; Chen and Nunez, 2010).

Toll-like receptors and other PRRs are found on many cell types throughout the body. However, hidden self-antigen mediated activation of such receptors on specific sentinel cells, particularly macrophages, triggers the initial release of pro-inflammatory cytokines, such as IL-1β. This cytokine response results in rapid activation of local blood vessel endothelial cells, resulting in recruitment of leukocytes from the blood stream, particularly neutrophils and monocytes. These phagocytic cells remove dead cells and other debris in the injured tissue, as well as performing a variety of other functions that promote tissue repair (Chen and Nunez, 2010).

In the case of brain injury, substantial evidence suggests that the PRR-expressing sentinel cells that initially trigger the injury-associated inflammatory response are brain tissue-resident macrophages (microglia) and astrocytes. The pro-inflammatory cytokines produced by microglia and astrocytes then initiate endothelial cell activation and recruitment of blood leukocytes (Fitch and Silver, 2008; Whitney et al., 2009), analogous to the inflammatory cascade that occurs in other tissues throughout the body. Although post-TBI inflammation plays an essential role in the healing response, there is also much evidence that the inflammatory response contributes to the death of bystander cells in the brain tissue that were not directly damaged by TBI (Loane and Byrnes, 2010). Furthermore, aspects of the inflammatory response may favor the formation of scar tissue (i.e., the glial scar), while disfavoring neuroregeneration (Fitch and Silver, 2008; Whitney et al., 2009; Griffiths et al., 2010; Neher et al., 2011). Thus, a detailed understanding of the inflammatory response to TBI is a necessary component in the effort to formulate successful strategies to diminish bystander cell injury and to promote neuroregeneration.

One of the gaps in the understanding of the biological response to TBI is how the magnitude of the injury influences the qualitative, quantitative, and kinetic characteristics of the inflammatory response. A recently published study using a rat model of TBI (in which members of our team participated) included the interesting finding that specific inflammatory mediators were produced in significant amounts in response to craniotomy. This study assessed a small collection of cytokine protein levels at days+1 and +7 post-injury, comparing tissues from craniotomy vs. naïve animals (Cole et al., 2011). However, as there was no comparison to a more severe brain injury in this study, it was not possible to determine how the observed cytokine levels induced by craniotomy compare to the cytokine response to more severe injury of the parenchyma.

Therefore, to extend the findings of this previous report, we initiated a study in mice to determine to what degree the magnitude and kinetics of the inflammatory response to severe and mild brain injury differ (note that the inflammatory response to TBI in mice and rats is highly similar Natale et al., 2003). To address this question, we performed histological, behavior, protein, and global gene expression analyses comparing a model of severe parenchymal injury [controlled cortical impact (CCI); Lighthall, 1988] to mild brain injury (craniotomy). Our data show that both severe and mild brain injury induce a qualitatively similar inflammatory response, involving highly overlapping sets of genes. However, kinetic analysis revealed that the inflammatory response to mild brain injury is of much shorter duration than the response to severe TBI, allowing severe- and mild-TBI to be readily discriminated at day +10 post-injury and beyond. These data therefore have implications for the diagnosis of TBI severity. Specifically, the development of accurate diagnostic tests of TBI severity that are based on imaging or biomarker analysis of the inflammatory response may require repeated measures over at least a 10-day period, post-injury.

## Materials and Methods

### Animals and surgical procedures

Ten- to twelve-week old C57BL/6 male mice were subjected to TBI using an Impact One™ Stereotaxic Impactor (myNeuroLab, St. Louis, MO, USA). Briefly, mice were anesthetized with 2% isoflurane in 98% oxygen, and were then positioned in the stereotaxic frame. Craniotomy was performed by a single skilled surgeon using a hand-held 5 mm trephine over the motor cortex (1.8 mm medial-lateral, 2 mm from Bregma). Mice were then subjected to CCI using a 3 mm flat-tip with a velocity of 5 m/s, a depth of 2.0 mm, and a duration of 200 ms. After trauma, the craniotomy was closed with the previously removed bone and bone wax, and the incision was closed with sutures. Craniotomy animals underwent the same procedure as the CCI group, except that the stereotaxic impactor was not used. Craniotomies were performed with great care, in order to avoid disruption of the dura. A few mice in the CCI group displayed slight hemorrhage, primarily on days +1 and +3 post-CCI. When hemorrhage was present, the wound was cleaned prior to tissue harvest. There was no visible hemorrhage in any craniotomy-only animal. Naïve mice were anesthetized with 2% isoflurane in 98% oxygen, monitored until recovery from anesthesia, and transferred to fresh cages. The Institutional Animal Care and Use Committee at Uniformed Services University (USU) approved all animal procedures.

### Histology

For hematoxylin and eosin (H&E) staining, brains were harvested from CCI, craniotomy, and naïve animals at day +7. Brains were perfused with 1 × PBS then fixed and stored in 4% paraformaldehyde. Tissue embedding, processing, and H&E staining were performed by Histoserv Inc. Note that the dura was lost from some regions of craniotomy and naïve brains during sectioning, although it was intact at the time of tissue harvest. Histology slides were viewed and scanned using the Nanozoomer Digital Pathology version 2.0-RS (Hamamatsu Photonics, Japan). Nanozoomer data were analyzed using NDP viewer software (Hamamatsu Photonics, Japan).

For immunofluorescence microscopy analysis, mice were perfused with a cold solution of 4% paraformaldehyde in 1 × PBS, followed by immediate brain harvest and 8–10 h cryopreservation in 30% sucrose. Brains were then frozen in Tissue-Tek OCT (Sakura Finetek, Torrance, CA, USA) and stored at −80°C. Coronal cryosections (20 μm) were collected and stored at −80°C until immunostaining with the anti-GFAP antibody.

### Tissue harvesting and RNA extraction

Animals were sacrificed on days +1, +2, +3, +7, +10, and +21. Mice were perfused with 1 × PBS and brains were collected. Two brain regions were harvested from naïve, craniotomy and CCI mice: a 5 mm diameter punch biopsy encompassing the exact injury site on the left hemisphere and another 5 mm biopsy recovering the equivalent non-injured (contralateral) site on the right hemisphere. The depth of the punch was approximately 5 mm, penetrating the base of the brain. RNA was extracted from the biopsy tissue using guanidinium isothiocyanate-phenol extraction (Chomczynski and Sacchi, 1987).

### Real-time PCR analyses

The above RNA samples (2 μg) were reverse transcribed to cDNA, using random hexamers and Superscript II Reverse Transcriptase (Life Technologies, Carlsbad, CA, USA), in a 1-h reaction at 42°C. Real-time PCR analysis of cDNA was performed using an RT-PCR master mix for TaqMan assays (SydLabs, Inc., Malden, MA, USA) and an iQ5 instrument (Bio-Rad, Hercules, CA, USA) in 96-well format with 20 μl reaction volume per well. Primers for TaqMan assays were designed using Primer Express 3.0 software (Life Technologies, Carlsbad, CA, USA). PCR primers and FAM-ZEN double-quenched probes were purchased from IDT (Coralville, IA, USA). Primer sequences are listed in Table 1. GAPDH was used as a normalization control for all probe sets. Samples were collected from both the ipsilateral and contralateral sites. Three or four mice were used for each experimental group at each time point.

**Table 1:** **Real-time PCR primers**.

**CCL2**	
Sense	GGCTCAGCCAGATGCAGTTAA
Anti-sense	CCTACTCATTGGGATCATCTTGCT
Probe	CCCCACTCACCTGCTGCTACTCATTCA
**IL-1β**	
Sense	GAGCACCTTCTTTTCCTTCATCTT
Anti-sense	CACACACCAGCAGGTTATCATCA
Probe	AGAAGAGCCCATCCTCTGTGACTCATGG
**TNF-α**	
Sense	GGTCCCCAAAGGGATGAGAA
Anti-sense	TGAGGGTCTGGGCCATAGAA
Probe	TTCCCAAATGGCCTCCCTCTCATCA
**AQP4**	
Sense	GGTTGGAGGATTGGGAGTCA
Anti-sense	GTGAACACCAACTGGAAAGTGATT
Probe	CACGGTTCATGGAAACCTCACCGC
**VIMENTIN**	
Sense	GGAGATGCTCCAGAGAGAGGAA
Anti-sense	GTGCCAGAGAAGCATTGTCAAC
Probe	CGAAAGCACCCTGCAGTCATTCAGACA
**MMP3**	
Sense	TGATGAACGATGGACAGAGGAT
Anti-sense	AGCCTTGGCTGAGTGGTAGAGT
Probe	TTGCTGCTCATGAACTTGGCCACTCC
**SAA3**	
Sense	CGCAGCACGAGCAGGAT
Anti-sense	GCTGTCAACTCCCAGGATCAA
Probe	AGCCTTCCATTGCCATCATTCTTTGCA
**C3**	
Sense	GCCAAGGACTGCAGACTGAAC
Anti-sense	CACTTCCGAAGACCCTTGTCA
Probe	CGCCGCCGTCGCTCAGTACAGT
**GAPDH**	
Sense	TGTGTCCGTCGTGGATCTGA
Anti-sense	CCTGCTTCACCACCTTCTTGA
Probe	CCGCCTGGAGAAACCTGCCAAGTATG

*Sequences of primers (5′ to 3′) used for TaqMan real-time PCR are listed. All hydrolysis probes were FAM-labeled and included an internal ZEN quencher. Each PCR amplicon crosses a splice junction*.

The delta Ct (ΔC_t_) method was used for PCR array data analysis. The normalized ΔC_t_ for each gene of interest (GOI) was calculated by deducting the C_t_ of the housekeeping gene (HKG: GAPDH) from the C_t_ of each GOI: $\text{Δ}{\text{C}}_{\text{t}}=\left({\text{C}}_{\text{t}}^{\text{GOI}}-{\text{C}}_{\text{t}}^{\text{HKG}}\right)$. The ΔΔCt for each GOI was calculated by deducting the average ΔC_t_ of GOI in the naïve or craniotomy group from the ΔC_t_ of each GOI in the CCI group: ΔΔC_t_ = ΔC_t_ (CCI group) – average ΔC_t_ (naïve or craniotomy group). The fold-change of each GOI compared to the naïve or craniotomy group was calculated as: Fold-change = 2^(−ΔΔCt)^.

### Electrochemiluminescent immunoassay analysis of cytokines in brain homogenates

Brain homogenates were prepared from punch biopsies (5 mm diameter cannula) from the injury and contralateral sites. Tissue was weighed and homogenized in 10 volumes per weight of T-Per extraction buffer (Pierce Biotechnology, Rockford, IL, USA) with Halt protease Inhibitor (Pierce Biotechnology, Rockford, IL, USA) utilizing a Bioruptor UCD-200 ultrasonic disruptor (Diagenode, Sparta, NJ, USA), as previously described (Cole et al., 2011). Total protein concentrations were determined using a Bradford Protein Assay Kit (Bio-Rad, Hercules, CA, USA). Analyte levels of cytokines were measured using the Mouse Pro-inflammatory 7-Plex Ultra-Sensitive Kit (Meso Scale Discovery, Gaithersburg, MD, USA). During the protocol, plates were washed using the BioTek ELx405 Select automated liquid handling platform. Imaging of the plates was performed using a Sector 6000 Imager (Meso Scale Discovery, Gaithersburg, MD, USA). A standard curve for each analyte was curve-fitted, allowing determination of the concentration in pg cytokine/mL sample volume in each well, which was normalized to total protein input, yielding analyte amount, expressed as pg cytokine/mg total protein.

### Microarray analyses

MouseRef-8 v2.0 Expression BeadChips (Illumina Inc., San Diego, CA, USA) were used to measure relative levels of mRNA expression for over 19,000 unique genes. Preparation of cDNA, probe hybridization, and data collection were carried out at the Cleveland Clinic. Background subtracted, quantile normalized data were analyzed using GenomeStudio (Illumina Inc., San Diego, CA, USA) and GSEA (Broad Institute, Cambridge, MA, USA) software packages.

### Behavior studies

All behavior tests were performed on days −1, +1, +3, +7, +10, +14, and +21, relative to surgical procedures. Rotarod testing was performed as previously described (Vitali and Clarke, 2004). Briefly, mice were acclimated to the rotarod apparatus (Ugo Basile, Collegeville, PA, USA) for 60 s at a fixed speed of 5 rpm. After the adaptation phase, animals were placed on the rotarod and the acceleration was increased from 5 to 60 rpm within 180 s. Latency to fall from the accelerating rotarod and the reached speed were recorded for each mouse. Three trials were performed for each animal and the average was reported.

For balance beam testing, mice were placed on a narrow beam (0.5 cm) and trained to cross the beam for three consecutive days before the first test. On the testing day, the mice were placed on the beam and the time spent to cross and the number of foot slips occurring during the beam cross were recorded. Three trials were performed for each animal and the average was calculated.

### Statistical analyses

Behavior data and protein expression data were analyzed using one-way analysis of variance (ANOVA) for multiple comparisons with *Tukey’s post hoc test*. A two-tailed Student’s *t*-test was used for comparison between two groups. Real-time PCR data were analyzed using a Mann–Whitney test. A *p* value < 0.05 was considered statistically significant.

## Results

### Histological analysis of brain injuries

**Figure 1:**
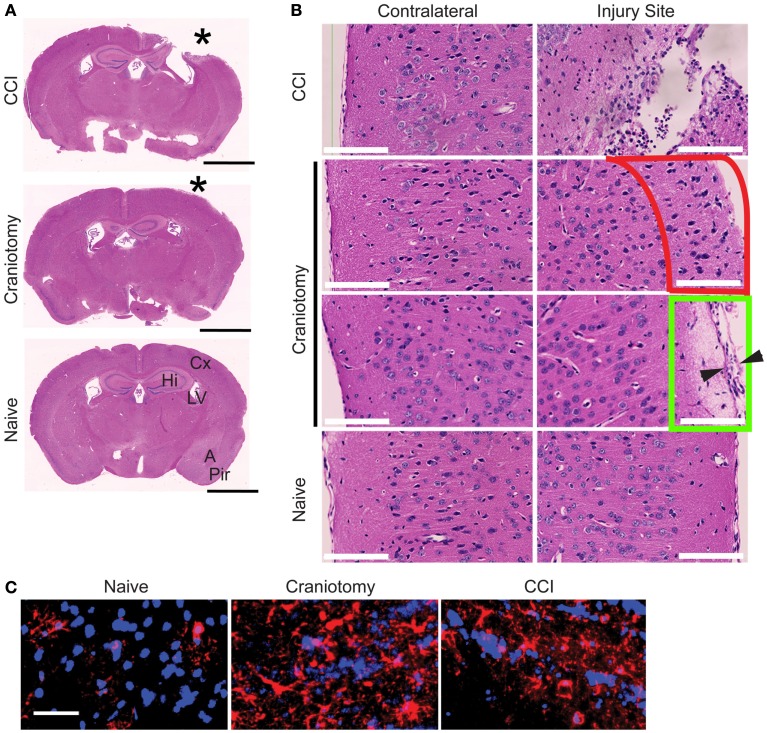
**Both severe and mild brain injury is accompanied by a cellular inflammatory response**. **(A)** H&E staining of coronal sections prepared from brains of CCI, craniotomy, or naïve animals at day +7. Images were collected on a Nanozoomer instrument and are displayed at a 2 × magnification. *, injury site. Bar, 2 mm. Cx, cortex; Hi, hippocampus; LV, lateral ventricle; A, amygdala; Pir, piriform cortex. **(B)** Tissue sections from **(A)** are shown at a 40 × magnification at the injury site or the equivalent contralateral location, as indicated. Red outline indicates cortical layer 1, and Green outline indicates region of altered dura (arrowheads) and underlying tissue at the injury site of the craniotomy samples. Bar, 100 μm. **(C)** Anti-GFAP immunofluorescence microscopy analysis of frozen brain sections from CCI, craniotomy, and naïve animals. Regions imaged were proximal to the injury site. CCI and craniotomy brains were harvested at day +3. Data are representative of three mice from each experimental group (naïve, craniotomy, and CCI). Bar, 40 μm.

Hematoxylin and eosin staining of coronal sections of brains from CCI, craniotomy, and naïve animals was performed to assess tissue damage. Low-magnification images revealed severe damage to the parenchyma of CCI brains, but no obvious tissue disruption in craniotomy animals (Figure 1A). Higher magnification H&E images showed immune cell infiltration and/or microglia activation and expansion around the site of injury, in response to the severe CCI injury (Figure 1B). Moreover, at the injury site in craniotomy animals, H&E staining suggested immune cell infiltration and/or expansion of activated microglia in cortical layer 1 (outlined in red), with disruption of the normal architecture of the dura and underlying cortical cells (outlined in green. Compare to contralateral sections; Figure 1B). Additionally, immunofluorescence microscopy using an anti-GFAP antibody showed evidence of astrogliosis near the site of injury in both CCI and craniotomy animals. Specifically, CCI and craniotomy animals had regions of high astrocyte density and enlarged astrocyte bodies, relative to naïve controls (Figure 1C). Together, the data in Figure 1 provide evidence that CCI and craniotomy-induced severe injury and mild injury to the brain, respectively. Additionally, this histological analysis provided evidence of a cellular inflammatory response following both CCI and craniotomy.

### Behavioral analysis of CCI and craniotomy animals

**Figure 2:**
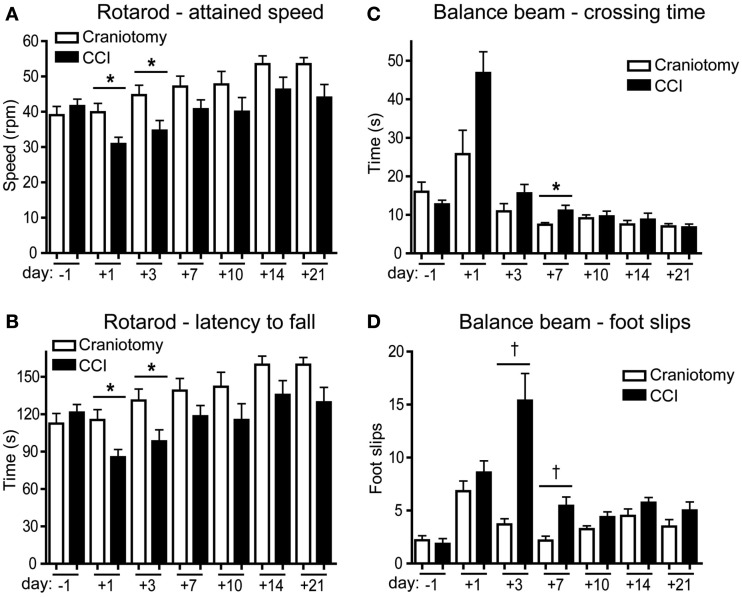
**Both severe and mild injury induce behavioral deficits**. **(A,B)** Rotarod assay (*n* = 12). Mice were assessed to determine the maximum speed attained **(A)** and the latency to fall **(B)** during execution of the accelerating rotarod task **(B)**. **(C,D)** Balance beam assay. Mice were assessed for latency to cross the beam **(C)** and the number of foot slips during the cross **(D)**. All tests were performed at day −1, and at multiple time points post-injury over a 21-day period. Error bars are SEM. The Student’s *t*-test was used to determine significance when comparing CCI to craniotomy samples; (* = *p* < 0.05, ^†^ = *p* < 0.005). Significant differences within the CCI and craniotomy groups were determined using ANOVA with Tukey’s multiple comparison test (Table 2).

CCI injury and craniotomy were performed directly over the motor cortex. Post-injury motor function was assessed via rotarod and balance beam assays (*n* = 12). In the rotarod task, CCI mice showed a significant deficit in performance on day +1, compared to pre-injury performance on day −1 (Table 2). Moreover, Figures 2A,B shows that both the maximum speed attained and the latency to fall from an accelerating rotarod decreased significantly among the CCI mice compared to the craniotomy mice at days +1 and +3. Although not statistically significant after day +3, there was a clear and consistent difference between the CCI and craniotomy animals, which persisted for at least 3 weeks post-injury (study end). Indeed, craniotomy mice showed no deficit following surgery, but rather continued to improve their performance throughout the first 2 weeks following injury [naïve mice show a very similar learning-based improvement over the same interval (data not shown)]. By day +7, the performance of CCI mice returned to the baseline level, and this improvement was statistically significant when comparing day +1 to day +14 (Table 2).

**Table 2:** **Statistically significant behavior data**.

Assay	*p* Values
**ROTAROD, ATTAINED SPEED**	
CCI day −1 vs. CCI day +1	*p <* 0.05
CCI day +1 vs. CCI day +14	*p <* 0.05
**ROTAROD, LATENCY TO FALL**	
CCI day −1 vs. CCI day +1	*p <* 0.05
CCI day +1 vs. CCI day +14	*p <* 0.05
**BALANCE BEAM, CROSSING TIME**	
Craniotomy day +1 vs. craniotomy day +3	*p <* 0.05
Craniotomy day +1 vs. craniotomy day +7	*p <* 0.01
Craniotomy day +1 vs. craniotomy day +10	*p <* 0.05
CCI day −1 vs. CCI day +1	*p <* 0.001
CCI day +1 vs. CCI day +3	*p <* 0.001
CCI day +1 vs. CCI day +7	*p <* 0.001
CCI day +1 vs. CCI day +10	*p <* 0.001
CCI day +1 vs. CCI day +14	*p <* 0.001
CCI day +1 vs. CCI day +21	*p <* 0.001
**BALANCE BEAM, FOOT SLIPS**	
Craniotomy day −1 vs. craniotomy day +1	*p <* 0.001
Craniotomy day +1 vs. craniotomy day +3	*p <* 0.01
Craniotomy day +1 vs. craniotomy day +7	*p <* 0.001
Craniotomy day +1 vs. craniotomy day +10	*p <* 0.01
CCI day −1 vs. CCI day +1	*p <* 0.05
CCI day −1 vs. CCI day +3	*p <* 0.001
CCI day +1 vs. CCI day +3	*p <* 0.01
CCI day +3 vs. CCI day +7	*p <* 0.001
CCI day +3 vs. CCI day +10	*p <* 0.001
CCI day +3 vs. CCI day +14	*p <* 0.01
CCI day +3 vs. CCI day +21	*p <* 0.01

*Behavior data in Figure 2 were analyzed by ANOVA with Tukey’s multiple comparison test. Significant differences between groups (*p <* 0.05) are shown. All comparisons not shown in this table were not significant, with the exception of significant CCI vs. craniotomy *t-*test data (included in Figure 2)*.

In the balance beam task, both CCI and craniotomy mice were significantly affected during the first week post-injury (Figures 2C,D). The crossing time was significantly increased for CCI animals, comparing day −1 to day +1, and the number of foot slips was significantly increased on days +1 and +3 (Table 2). For the craniotomy animals, only the number of foot slips was significantly increased between day −1 and +1 (Table 2). Notably, during the first week post-injury, motor performance was more severely impaired by CCI than by craniotomy. These data were significant for the beam crossing time on day +7 and for foot slips on days +3 and +7 (Figures 2C,D). By day +10, both craniotomy and CCI mice showed a significant improvement in their performance on the balance beam, vs. the post-injury day of most severe impairment (Figures 2C,D; Table 2).

Together, the data in Figure 2 show that the effect of brain injury on motor function was most pronounced during the first week post-TBI, with some deficits in function observed for both CCI and craniotomy animals. The significant differences observed in the performance of CCI vs. craniotomy animals during the first week post-injury is consistent with the severe parenchymal damage induced by CCI vs. the more subtle brain injury induced by craniotomy (Figure 1).

### Inflammatory cytokine protein response to traumatic brain injury

Previous studies have established that moderate to severe TBI is accompanied by inflammation (Ciallella et al., 2002; Harting et al., 2008; Rhodes et al., 2009; Dalgard et al., 2012). A recent study by members of our team suggested that there is also significant induction of several inflammatory mediators in response to mild brain injury (craniotomy) in the rat model system (Cole et al., 2011). To determine whether mice also show similar inflammatory responses to both CCI and craniotomy, we profiled the protein expression of a subset of cytokines in brain tissue using a multiplexed ELISA detection platform. For this analysis, we employed a 5 mm punch biopsy to recover the tissue at the site of CCI and craniotomy, and from the equivalent site at the non-injured (contralateral) hemispheres. Tissues were harvested from injured animals at days +1, +3, and +7, and from naïve mice.

**Figure 3:**
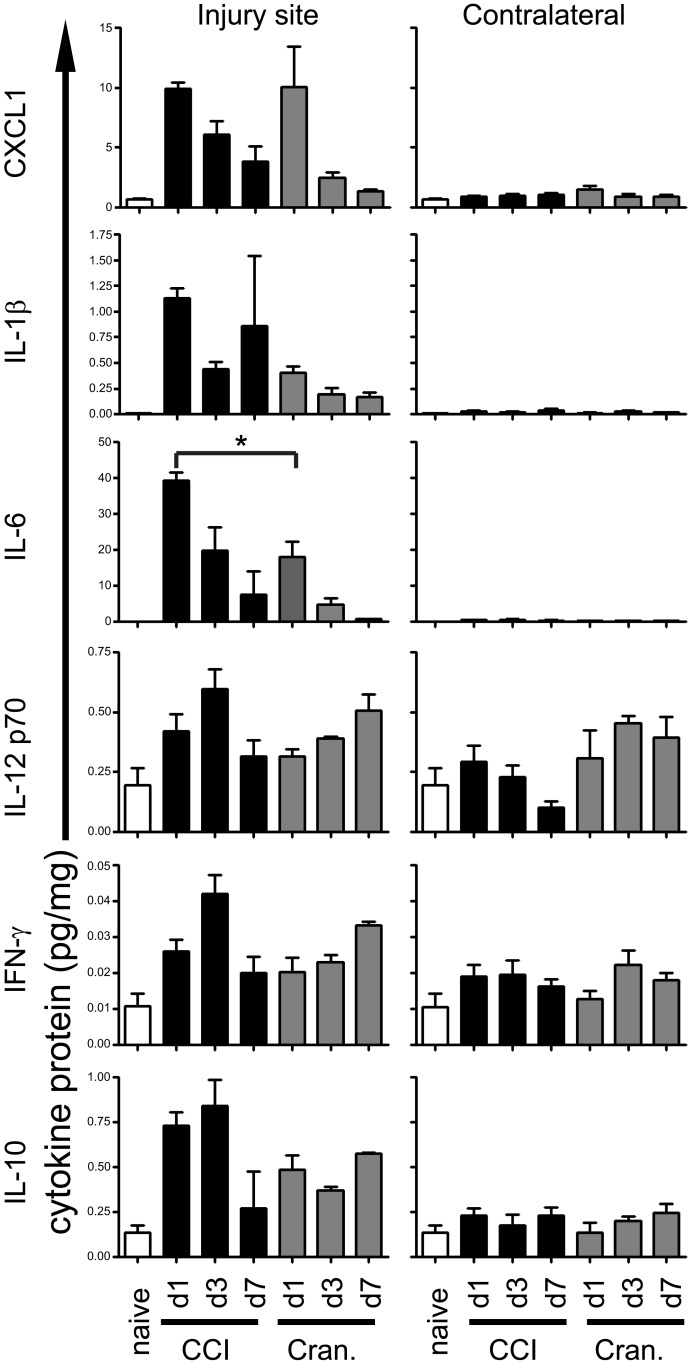
**Inflammatory cytokines are induced to similar levels by both severe and mild brain injury**. Brain tissue biopsies from the injured or contralateral sites were harvested from CCI and craniotomy (Cran.) animals on the indicated days post-injury. Tissues were also harvested from naïve animals to establish the baseline for each assay. Cytokine proteins in brain tissue homogenates were quantified by electrochemiluminescent immunoassay. Three mice from each experimental group (naïve, craniotomy, and CCI) were used for this analysis. Error bars are SEM. Statistically significant differences between groups are indicated in Table 3. Additionally, the single finding of a statistically significant difference between CCI and craniotomy on equivalent days is indicated by an asterisk (*).

Of the seven cytokines measured, six were significantly increased following CCI, as compared to naïve controls (Figure 3; Table 3). Of these six cytokines, peak expression was observed at day +1 for three cytokines (CXCL1, IL-1β, and IL-6), while the other three cytokines exhibited peak expression at day +3 (IL-12p70, IFN-γ, and IL-10).

**Table 3:** **Statistically significant changes in cytokine protein levels**.

Cytokine measurement	*p* Values
**CXCL1 – INJURY SITE**	
Naive vs. CCI day +1	*p <* 0.001
Naive vs. CCI day +3	*p <* 0.05
Naive vs. craniotomy day +1	*p <* 0.001
Craniotomy day +1 vs. craniotomy day +3	*p <* 0.01
Craniotomy day +1 vs. craniotomy day +7	*p <* 0.01
**CXCL1 – CONTRALATERAL SITE**	
Naive vs. craniotomy day +1	*p <* 0.01
**IL-6 – INJURY SITE**	
Naive vs. CCI day +1	*p <* 0.001
Naive vs. CCI day +3	*p <* 0.01
Naive vs. craniotomy day +1	*p <* 0.05
CCI day +1 vs. CCI day +3	*p <* 0.05
CCI day +1 vs. CCI day +7	*p <* 0.001
CCI day +1 vs. craniotomy day +1	*p <* 0.05
**IL-1β** **– INJURY SITE**	
Naive vs. CCI day +1	*p <* 0.05
**IL-10 – INJURY SITE**	
Naive vs. CCI day +1	*p <* 0.01
Naive vs. CCI day +3	*p <* 0.001
Naive vs. craniotomy day +7	*p <* 0.05
CCI day+3 vs. CCI day +7	*p <* 0.05
**IL-12 p70 – INJURY SITE**	
Naive vs. CCI day +3	*p <* 0.01
Naive vs craniotomy day +7	*p <* 0.05
**IFNγ** –**INJURY SITE**	
Naive vs. CCI day +3	*p <* 0.001
Naive vs. craniotomy day +7	*p <* 0.01
CCI day +3 vs. CCI day +7	*p <* 0.05

*Cytokine data in Figure 3 were analyzed by ANOVA with Tukey’s multiple comparison test. Significant differences between groups (*p < *0.05) are shown. All comparisons not shown were not significant*.

In comparing naïve animals to CCI animals at the injury site, CXCL1 and IL-6 were significantly increased at days +1 and +3, while IL-1β was significantly increased only at day +1. The craniotomy tissue demonstrated a similar significant increase in CXCL1 and IL-6 protein expression at day +1, in comparison to naïve tissue. The contralateral site did not exhibit significant increases in production of these cytokines in CCI or craniotomy subjects. The exception was CXCL1, which was significantly increased in craniotomy subjects at day +1, as compared to controls.

In contrast to CXCL1, IL-1β, and IL-6, the changes in peak expression for IL-12p70, IFN-γ, and IL-10 after injury were delayed and modest. Significant increases were detected only at the injury site. When comparing injured to naïve tissue, IL-12p70, IFN-γ, and IL-10 were all significantly elevated at day +3, while only IL-10 was significantly elevated at day +1. After day +3, expression of these cytokines declined to non-significant levels, vs. naïve. Interestingly, the kinetics of expression of these cytokines in craniotomy tissue was slower than in CCI tissue, with significant increases of IL-12p70, IFN-γ, and IL-10 not observed until day +7.

Together, the data in Figure 3 and Table 3 illustrate that expression of six different cytokines was significantly increased in response to severe brain injury (CCI). Importantly, expression of all but one of these cytokines was also significantly increased at the injury site in response to mild brain injury (craniotomy). Additionally, the peak levels of cytokine production were similar (within a factor of three), when comparing CCI vs. craniotomy at the injury site. Indeed, when comparing each day for CCI to the corresponding day for craniotomy, the only significant difference was IL-6 at day +1 (Figure 3; Table 3). Thus, severe brain injury and mild brain injury induce a quantitatively similar inflammatory cytokine response during the first 7 days post-injury.

### Microarray analysis of inflammatory gene expression

**Figure 4:**
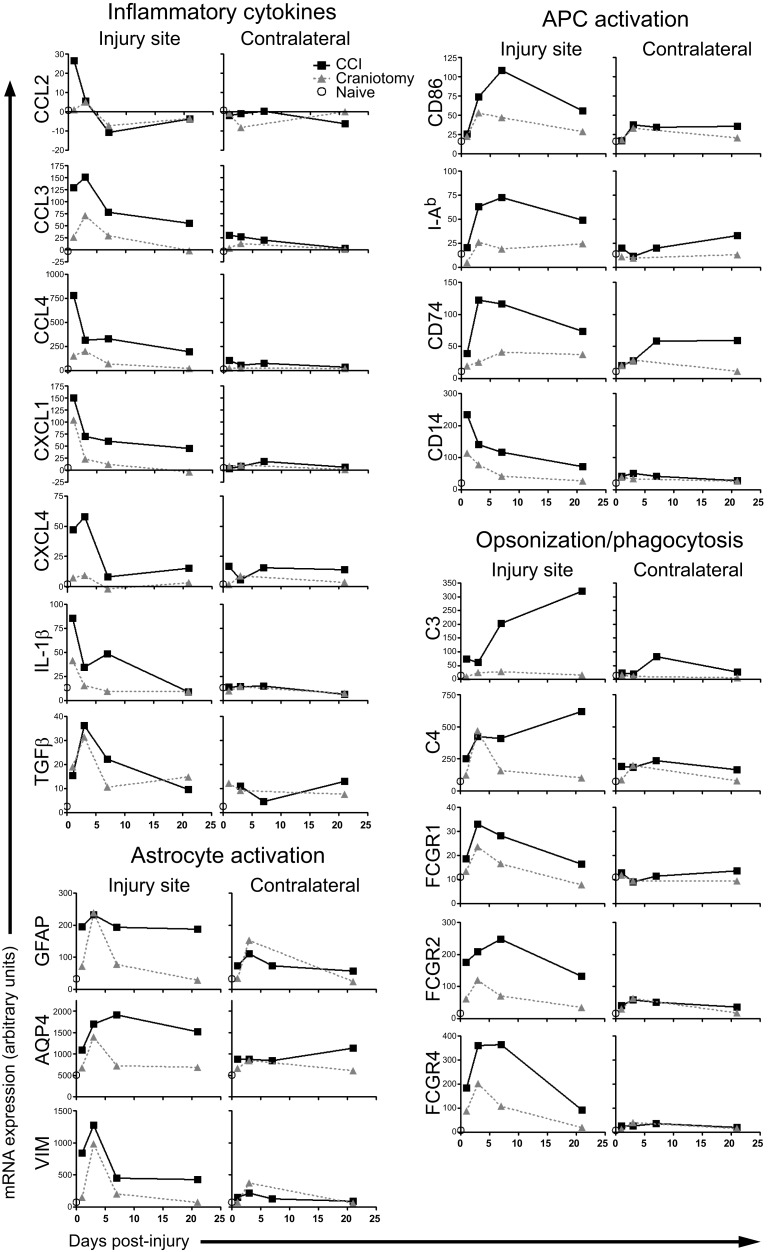
**Microarray analysis shows severe and mild brain injury induce expression of multiple inflammation-related transcripts with similar kinetics**. Injury site and contralateral site biopsies were harvested from CCI and craniotomy mice at the indicated times post-injury. Brain tissue was also harvested from naïve animals. Total RNA samples from individual animals from each experimental group (3–4 mice/group) were pooled, and cDNA was synthesized and analyzed by microarray. Relative expression levels are shown for selected genes in the indicated functional groups.

To assess whether the above expression data for selected inflammatory proteins could be generalized to the global inflammatory response, we performed a genome-wide microarray analysis. Brain tissue biopsies were collected from CCI, craniotomy, and naive animals, as described for the cytokine protein analysis in Figure 3. mRNA was harvested from three to four animals per time point per condition, and samples from individual animals were pooled prior to cDNA synthesis. Pooled cDNAs were analyzed via Illumina bead-chip microarrays. We examined selected markers of inflammation to assess general trends in inflammatory gene expression. We chose genes from four diverse functional sets: inflammatory cytokines, astrocyte activation markers, markers of antigen presenting cell (APC)/microglia activation, and effectors of opsonization and phagocytosis. Although a number of different kinetic patterns were noted, a consistent observation was that the genes induced by CCI were also induced by craniotomy (Figure 4). Interestingly, the general gene expression kinetic trends were quite similar in the CCI and craniotomy groups, with a day +1 or +3 expression peak frequently observed in both groups. For both CCI and craniotomy groups, induction of inflammatory gene expression in the contralateral sample was either not detected or less than that observed in the CCI tissue. However, a general difference between the CCI and craniotomy groups was that values generally returned to baseline in the craniotomy group by day +21. In contrast, the CCI tissues generally remained above the naïve baseline at day +21. Thus, these data show that inflammation-related gene expression is highly similar between severe parenchymal injury (CCI) and mild brain injury (craniotomy), with regard to the intensity of gene expression and the kinetic pattern of gene expression. The major notable difference was the persistence of inflammation in response to severe injury (CCI) at day +21, in apparent contrast to the mild injury (craniotomy) group.

### Gene set enrichment analysis of microarray data

We also analyzed the microarray data by Gene Set Enrichment Analysis (GSEA; Subramanian et al., 2005) to determine whether the patterns of inflammatory gene expression among genes sampled in Figure 4 were representative of the global inflammatory response to CCI and craniotomy. For this analysis, we chose CCL3 as a representative phenotype among the gene expression profiles shown in Figure 4. GSEA software identified gene expression profiles with similarity to CCL3 and clustered these profiles into functionally related gene sets. Over 200 gene sets were scored as enriched, and approximately 100 of these sets were scored as statistically significant.

**Figure 5:**
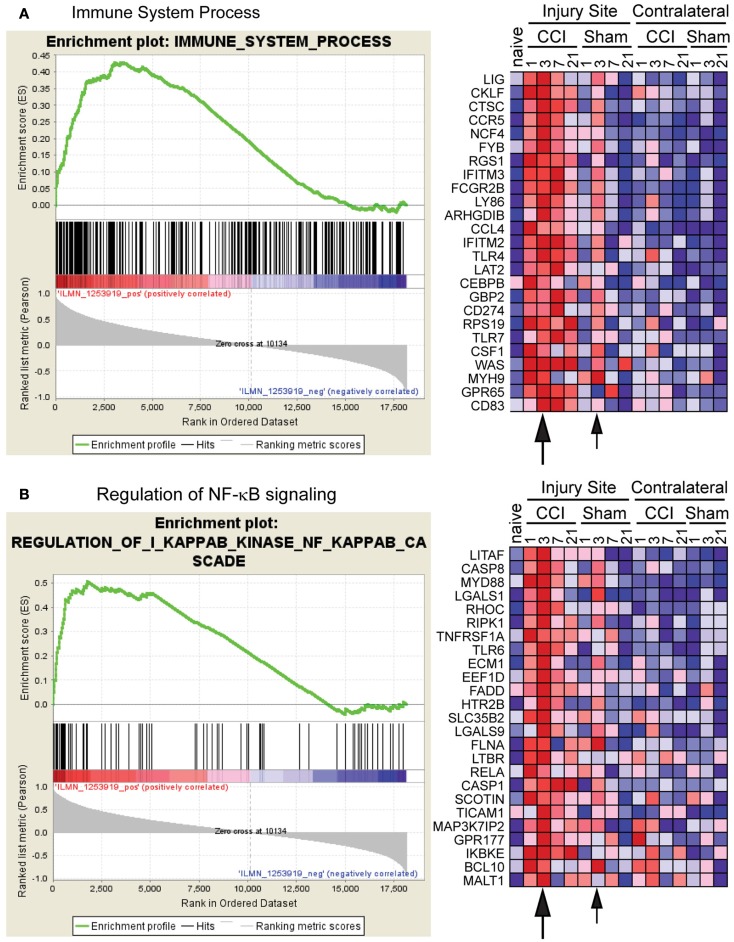
**Gene set enrichment analysis (GSEA) confirms induction of a broad inflammatory response by both severe and mild injury**. Microarray data were analyzed using GSEA software to identify functionally related groups of genes (gene sets) with statistically significant enrichment, using CCL3 as the gene expression phenotype. The figure shows the enrichment plot and the top 25 enriched genes for **(A)** the Immune System Process set and **(B)** the Regulation of NF-κB Signaling (Regulation of IκB kinase/NF-κB Cascade) set. The plot on the left shows the distribution of genes in the set that are positively and negatively correlated with the CCL3 phenotype. The plot on the right shows the relative gene expression (red = high, blue = low) for each gene for the indicated samples. Note that the overall kinetic profiles are similar for the CCI and craniotomy (Cran) samples, with a prominent gene expression peak at day +3 (large and small arrows indicate the day +3 peak for CCI and craniotomy, respectively). However, the craniotomy samples generally show a lower intensity of gene expression, particularly at day +21.

Among the sets with highest statistical significance were the Immune System Process set and the Regulation of IκB Kinase/NF-κB Cascade set, both of which reflect components of the inflammatory response (Figure 5). For these two sets, the false discovery rate (FDR) *q*-values were 0.082 and 0.115, respectively (values < 0.25 are considered significant Subramanian et al., 2005). For both sets, similar trends in gene activation were observed between CCI and craniotomy animals, with a day +3 activation peak being most prominent for both CCI (large arrow) and craniotomy (small arrow). Activation was strong at the injury site, but not detected or weak at the contralateral site, consistent with the analysis of selected genes in Figure 4. However, such differences in magnitude of activation appeared greatest at day +21, at which point the CCI-induced RNAs generally remained elevated relative to naïve; whereas the craniotomy-induced RNAs had generally returned to the naïve baseline value.

### Real-time PCR analysis of inflammatory gene expression kinetics

**Figure 6:**
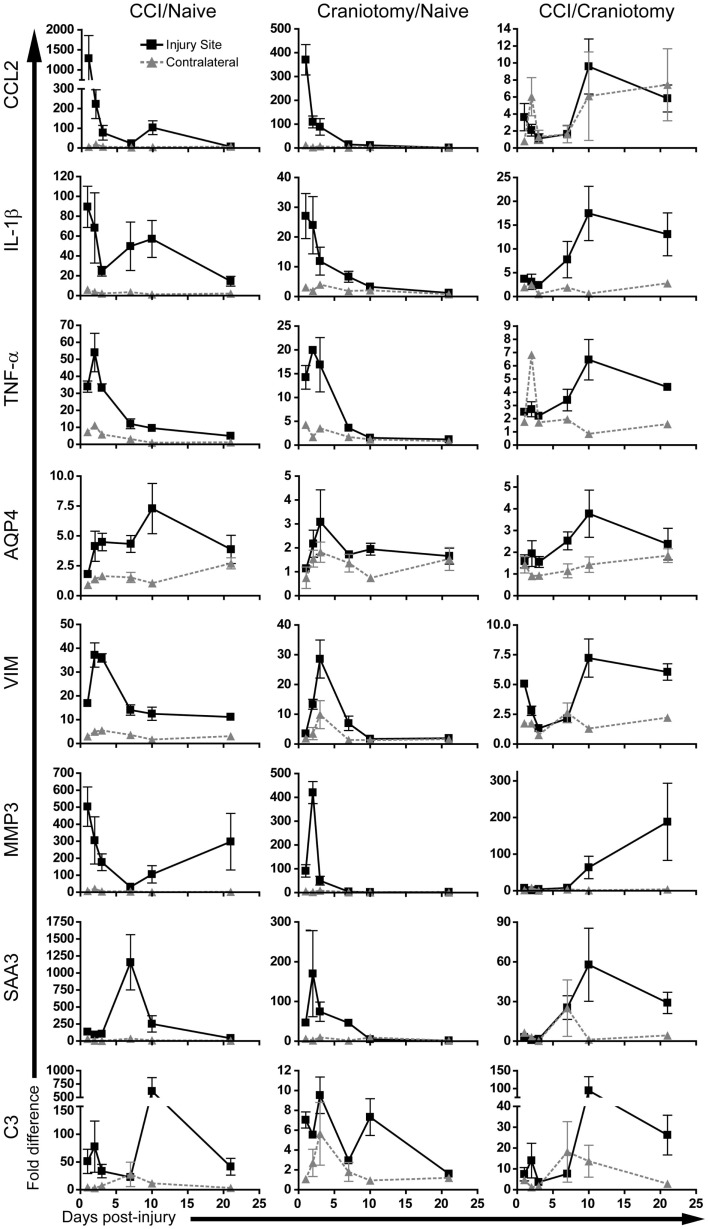
**The inflammatory response to severe brain injury persists for several weeks, whereas the response to mild injury declines rapidly after day +7**. Real-time PCR analysis of mRNA levels for the indicated genes at days +1, +2, +3, +7, +10, and +21. Samples from individual animals from both ipsilateral and contralateral sites were tested for the expression of the specified genes. Three or four mice were analyzed for each time point in each experimental group. Data are expressed as ratios of CCI/Naïve, Craniotomy/Naïve, and CCI/Craniotomy, as indicated, with the *y*-axis indicating fold-difference in gene expression. Note that the CCI/Craniotomy fold-difference at the injury site is greatest after day +7 for all analyzed genes. Significant differences in mRNA levels in the CCI vs. Craniotomy groups are listed in Table 4. Error bars are SEM.

The above microarray data suggested that CCI and craniotomy induce a highly similar inflammatory gene expression program in the brain. Moreover, these data suggested that the magnitude of inflammation-associated gene expression was similar at all time points through day +7. However, the data also suggested that the inflammatory gene expression in the CCI and craniotomy groups diverge by day +21, with robust inflammation maintained by the CCI animals, but not the craniotomy animals. To confirm these observations with a more sensitive technique, we used TaqMan real-time PCR to quantify relative levels of transcription for an assortment of eight inflammation-associated genes (Figure 6; Table 4), which included cytokines (CCL2, IL-1β, TNF-α), markers of astrocyte activation [Aquaporin-4 (AQP4), Vimentin (VIM), Matrix metalloproteinase-3 (MMP3)], and RNAs encoding proteins of the macrophage acute phase response [serum amyloid A3 (SAA3), complement C3]. In addition to the days +1, +3, +7, and +21 time points analyzed in the microarray data, we also included days +2 and +10. This real-time PCR analysis was performed on samples from individual mice (3–4 mice/condition/time point), allowing quantification of biological variability.

**Table 4:** **Statistically significant changes in gene expression**.

Assay	*p* Values
**CCL2 – INJURY SITE**	
CCI vs. craniotomy – day+10	*p <* 0.05
CCI vs. craniotomy – day+21	*p <* 0.05
**IL-1β** **– INJURY SITE**	
CCI vs. craniotomy – day+1	*p <* 0.05
CCI vs. craniotomy – day+10	*p <* 0.05
**TNFα – INJURY SITE**	
CCI vs. craniotomy – day+1	*p <* 0.05
CCI vs. craniotomy – day+10	*p <* 0.05
CCI vs. craniotomy – day+21	*p <* 0.05
**AQP4 – INJURY SITE**	
CCI vs. craniotomy – day+7	*p <* 0.05
CCI vs. craniotomy – day+10	*p <* 0.05
CCI vs. craniotomy – day+21	*p <* 0.05
**VIM – INJURY SITE**	
CCI vs. craniotomy – day+1	*p <* 0.0001
CCI vs. craniotomy – day+2	*p <* 0.05
CCI vs. craniotomy – day+10	*p <* 0.05
CCI vs. craniotomy – day+21	*p <* 0.05
**MMP3 – INJURY SITE**	
CCI vs. craniotomy – day+1	*p <* 0.05
**SAA3 – INJURY SITE**	
CCI vs. craniotomy – day+10	*p <* 0.05
CCI vs. craniotomy – day+21	*p <* 0.05
**C3 – INJURY SITE**	
CCI vs. craniotomy – day+1	*p <* 0.05
CCI vs. craniotomy – day+7	*p <* 0.05
CCI vs. craniotomy – day+21	*p <* 0.05

*Real-time PCR data in Figure 6 were analyzed using a Mann–Whitney test. Significant differences between CCI and craniotomy groups (*p < *0.05) are shown. All comparisons not shown were not significant*.

Data were quantified as fold-difference in expression for three ratios: CCI/Naïve, Craniotomy/Naïve, and CCI/Craniotomy (Figure 6). This analysis generally provided a confirmation of microarray data presented in Figure 4. For example, in the case of IL-1β, the peak mRNA levels at the injury site were at day +1 for both CCI and craniotomy. Also, the IL-1β mRNA levels at the injury site for the CCI samples at day +1 was approximately 2 × higher by microarray vs. 4 × higher by real-time PCR, as compared to the craniotomy samples. For certain transcripts, such as CCL2, the real-time PCR provided much higher signal-to-noise than the microarray analysis, revealing a strong induction of CCL2 transcription by craniotomy that was not evident in the microarray data. Also, the inclusion of additional time points in the real-time PCR analysis revealed important details of the kinetics for the expression of certain genes. For example, day +10 represents the peak AQP4 and C3 mRNA expression (among included time points) at the injury site for CCI animals. Day +10 also represents a secondary peak of transcription of CCL2 and IL-1β genes, in response to CCI.

Importantly, the real-time PCR analysis confirmed the overall trend suggested by the microarray analyses (Figures 4 and 5). Specifically, at the site of injury, whereas inflammatory gene expression persisted beyond day +7 (and in some cases continued to intensify) in the CCI animals, inflammatory gene expression in the craniotomy animals generally reached or approached baseline values beyond day +7. This trend was particularly apparent when assessing the CCI/craniotomy ratios for the injury site data, in which the greatest difference between CCI and craniotomy was at day +10 or +21 for all eight mRNAs. Moreover, whereas the majority of transcripts exhibited less than a 10-fold difference in the CCI/craniotomy ratio between days +1 and +7, three genes (MMP3, SAA3, and C3) showed a greater than 50-fold difference between mRNA abundance in CCI and craniotomy animals at day +10 or +21 (note, however, that the MMP3 data were not significant, due to high variability at these late time points).

Notably, for all transcripts except for MMP3, statistically significant differences were observed between CCI and craniotomy tissues (at the injury site) for day +10 and/or +21. Although significant differences between these same groups were also seen at day +1 for five transcripts (IL-1β, TNFα, VIM, MMP3, and C3), it is important to note that the fold-differences were greater at day +10 and/or +21. Together, these data reinforce the conclusions suggested by Figures 3 and 5. Specifically, severe brain injury and mild brain injury induce very similar inflammatory responses through approximately day +7. Following day +7 (during the period of days +10 through +21 in our analysis), the inflammatory response to severe injury persists, whereas the response to mild injury returns to baseline for most genes.

## Discussion

In this study, we performed a 21-day kinetic analysis of the inflammatory response to severe and mild brain injury, examining both the injury site and the equivalent site on the contralateral hemisphere. The histological data in Figure 1 support the severe nature of the CCI injury and the more subtle nature of injury-associated with craniotomy. Specifically, CCI-induced a substantial loss of brain tissue beneath the injury site, accompanied by considerable inflammatory cell infiltration and astrocyte activation. In contrast, the craniotomy animals showed no evidence of tissue loss. However, craniotomy was not innocuous, as demonstrated by histological changes within the injury site: H&E staining of coronal sections showed increased numbers of inflammatory cells and changes to the dura and underlying parenchyma. Anti-GFAP immunofluorescence suggested astrogliosis. Thus, although there was no apparent brain tissue loss due to craniotomy, there was clear evidence of an inflammatory response.

Behavior data (Figure 2) showed a clear difference in impairment of motor function in CCI vs. craniotomy animals. Between day −1 and day +1, only the CCI animals exhibited a significant impairment in performance in the rotarod assay, whereas both injuries significantly impaired performance on the balance beam. Thus, both severe and mild injury to the motor cortex cause at least a transient functional impairment. However, direct comparison of CCI vs. craniotomy animals revealed significant differences on days +1 and +3 for the rotarod, and on days +3 and +7 for the balance beam. Based on these data, we conclude that craniotomy induces a mild and transient functional impairment, while CCI more severely impairs function for at least a week post-injury. The behavior data are consistent with the histological data (Figure 1).

Quantification of protein levels for a limited selection of inflammatory cytokines also supported the histological data. In comparison to naïve controls, there was a significant elevation of each of the measured inflammatory cytokines at one or more time points at the injury site in both the CCI and craniotomy groups (with the exception of IL-1β, for which the measured increase did not reach significance in the craniotomy group). Although increases in cytokine expression were generally confined to the site of injury, it is notable that in one instance (CXCL1 at day +1), we did detect a significant increase at the contralateral site in the craniotomy group. This finding suggests that certain cytokines diffuse over considerable distances following brain injury, and/or that long-range diffusion of hidden self-antigens stimulates resident pro-inflammatory cells (e.g., microglia and astrocytes) far from the site of injury. The mRNA expression data in Figures 4 and 6 are suggestive of the latter possibility.

Surprisingly, over the first week post-injury, the levels of inflammatory protein expression in the CCI and craniotomy groups were of similar magnitude, even though the extent of tissue damage was substantially different. Indeed, the only statistically significant difference between CCI and craniotomy was for IL-6 at day +1. These data suggest that major differences in the extent of brain tissue injury are reflected by modest differences in inflammatory cytokine production.

Because our cytokine protein analysis included a limited number of inflammatory mediators, we performed a genome-wide microarray analysis. The microarray data confirmed and extended the cytokine protein measurements. Specifically, these data showed that CCI and craniotomy induce the transcription of an identical or highly overlapping set of soluble and cell-associated regulators of inflammation. Furthermore, the kinetics and magnitude of induction of these genes was highly similar during the first week post-injury. Therefore, through day +7, there is little difference between the global inflammatory response induced by a severe brain lesion with substantial tissue destruction vs. a mild brain injury with minimal damage to the parenchyma.

Importantly, however, our data show that following day +7, the inflammatory responses to severe and mild brain injury become discordant. In general, inflammatory gene expression persisted thorough at least day +21 in the CCI group, while returning to naïve baseline levels in the craniotomy animals by day +10. We presume that this difference reflects both the time required for phagocytic cells to clear dead tissue and the ongoing cell death (and concomitant pro-inflammatory signaling by persistent release of hidden self-antigens) in the penumbral region surrounding the site of direct tissue damage (Fitch and Silver, 2008; Loane and Byrnes, 2010) in the severe injury (CCI) group. Not only did the CCI animals exhibit persistent (≥21 days) expression of the great majority of measured inflammation-associated genes, but a subset of genes, including complement C3, reached their peak expression *after* day +7.

In general, the mRNA and protein expression data are consistent with the behavior data: the greatest behavioral deficit correlated with the peak of the inflammatory response. Such a finding is consistent with the phenomenon of cytokine-induced sickness behavior, in which pro-inflammatory cytokines interact with the brain, inducing broad behavioral changes (Dantzer, 2001; Capuron and Miller, 2011). Based on our behavior data (which showed no significant behavioral deficit beyond day +7), it is not clear whether the persistent inflammatory response in CCI animals is correlated with any functional deficits. As the CCI injury resulted in clear tissue destruction, it is also difficult to assess the degree to which cell loss vs. inflammation contributed to the observed phenotypes. To better assess the relationship between inflammation and functional deficits, it will thus be important to develop TBI models that yield persistent inflammation with minimal tissue destruction, for testing with a wide array of behavioral assays.

We are not aware of detailed kinetic assessments of the global inflammatory response following other types of TBI, although the limited existing data are in general agreement with our findings. Specifically, a recent study using a mouse model of blast injury (Cernak et al., 2011) included semi-quantitative PCR findings consistent with our data. Measurements of CCL2 in the hippocampus and brainstem and GFAP in the hippocampus showed significant elevation of transcription in response to moderate blast, persisting until at least day +30 (study end). Mild blast also caused increased transcription of these genes, with day +1 levels very similar to moderate blast. However, by day +30, mRNA levels in the mild blast animals returned to baseline. Thus, the relationship between the inflammatory response induced by mild blast vs. moderate blast may be analogous to the relationship between craniotomy and CCI.

Regarding closed-head concussive injury models (weight drop or impactor device), investigators have reported transient increases in transcription of inflammatory genes (Crack et al., 2009; Israelsson et al., 2009) and persistent activation of microglia (Venkatesan et al., 2010). Another study failed to detect significant elevations of inflammatory cytokine proteins (Semple et al., 2010), although there were trends toward elevation of inflammatory mediators at early times post-injury. Because of differences in injury delivery, time points assessed, and analytical methods, it is difficult to distil these data to a consensus finding regarding inflammation following closed-head concussive injury. In general, however, these studies do suggest the induction of a transient inflammatory response, with peak expression by day +1 or day +3.

Although we speculate that repeated closed-head injury will trigger a more persistent inflammatory state in the brain, we are not aware of published data addressing this prediction. Given the accumulating clinical data showing striking pathology resulting from repeated concussive injuries (Baugh et al., 2012), it will be important to determine whether persistent inflammation contributes to the neurodegenerative response associated with repeated closed-head concussive injury.

With the increasing focus on TBI resulting from military deployments, concussion-prone sports, and auto accidents, many investigators are attempting to develop minimally invasive strategies to assess the extent and/or severity of brain damage resulting from a known or suspected recent TBI (Kubal, 2012). As inflammation is a predictable response to brain injury, measurement of inflammation is being explored as a proxy for brain damage. Specific approaches include the use of probes to detect activated macrophages in the brain through magnetic resonance imaging (MRI) and positron emission tomography (PET; Stoll and Bendszus, 2009; Wunder et al., 2009; Sibson et al., 2011), and use of antibody-based assays to detect biomarkers of inflammation in the blood or cerebral-spinal fluid (Agoston et al., 2009; Korfias et al., 2009; Svetlov et al., 2009). However, our data illustrate that such strategies, when employed as single time point tests within the first week of injury, may be unable to accurately assess the severity of brain tissue injury. Based on our data, we predict that accurate quantification of TBI severity will require repeated measures of inflammation performed over a period of at least 10 days. We furthermore propose that those inflammation-associated genes which show peak expression after day +7 may represent ideal biomarkers of TBI severity. This idea will require further validation.

## Conflict of Interest Statement

The views expressed are those of the authors and do not necessarily reflect those of the Uniformed Services University or the Department of Defense. The authors declare no competing financial interests.

## References

[B1] Agoston D. V. Gyorgy A. Eidelman O. Pollard H. B. (2009). Proteomic biomarkers for blast neurotrauma: targeting cerebral edema, inflammation, and neuronal death cascades. J. Neurotrauma 26, 901–911 10.1089/neu.2008.0724 19397421

[B2] Baugh C. M. Stamm J. M. Riley D. O. Gavett B. E. Shenton M. E. Lin A. (2012). Chronic traumatic encephalopathy: neurodegeneration following repetitive concussive and subconcussive brain trauma. Brain Imaging Behav. 6, 244–254 10.1007/s11682-012-9164-5 22552850

[B3] Capuron L. Miller A. H. (2011). Immune system to brain signaling: neuropsychopharmacological implications. Pharmacol. Ther. 130, 226–238 10.1016/j.pharmthera.2011.01.014 21334376 PMC3072299

[B4] Cernak I. Merkle A. C. Koliatsos V. E. Bilik J. M. Luong Q. T. Mahota T. M. (2011). The pathobiology of blast injuries and blast-induced neurotrauma as identified using a new experimental model of injury in mice. Neurobiol. Dis. 41, 538–551 10.1016/j.nbd.2010.10.025 21074615

[B5] Chen G. Y. Nunez G. (2010). Sterile inflammation: sensing and reacting to damage. Nat. Rev. Immunol. 10, 826–837 10.1038/nri2873 21088683 PMC3114424

[B6] Chomczynski P. Sacchi N. (1987). Single-step method of RNA isolation by acid guanidinium thiocyanate-phenol-chloroform extraction. Anal. Biochem. 162, 156–159 10.1006/abio.1987.9999 2440339

[B7] Ciallella J. R. Ikonomovic M. D. Paljug W. R. Wilbur Y. I. Dixon C. E. Kochanek P. M. (2002). Changes in expression of amyloid precursor protein and interleukin-1beta after experimental traumatic brain injury in rats. J. Neurotrauma 19, 1555–1567 10.1089/089771502762300229 12542857

[B8] Cole J. T. Yarnell A. Kean W. S. Gold E. Lewis B. Ren M. (2011). Craniotomy: true sham for traumatic brain injury, or a sham of a sham? J. Neurotrauma 28, 359–369 10.1089/neu.2010.1427 21190398 PMC3057208

[B9] Crack P. J. Gould J. Bye N. Ross S. Ali U. Habgood M. D. (2009). The genomic profile of the cerebral cortex after closed head injury in mice: effects of minocycline. J. Neural Transm. 116, 1–12 10.1007/s00702-008-0145-1 19018450

[B10] Dalgard C. L. Cole J. T. Kean W. S. Lucky J. J. Sukumar G. Mcmullen D. C. (2012). The cytokine temporal profile in rat cortex after controlled cortical impact. Front. Mol. Neurosci. 5:6 10.3389/fnmol.2012.00006 22291617 PMC3265961

[B11] Dantzer R. (2001). Cytokine-induced sickness behavior: mechanisms and implications. Ann. N. Y. Acad. Sci. 933, 222–234 10.1111/j.1749-6632.2001.tb05827.x 12000023

[B12] Fitch M. T. Silver J. (2008). CNS injury, glial scars, and inflammation: inhibitory extracellular matrices and regeneration failure. Exp. Neurol. 209, 294–301 10.1016/j.expneurol.2007.05.014 17617407 PMC2268907

[B13] Griffiths M. R. Gasque P. Neal J. W. (2010). The regulation of the CNS innate immune response is vital for the restoration of tissue homeostasis (repair) after acute brain injury: a brief review. Int. J. Inflam. 2010, 151097 21152121 10.4061/2010/151097 PMC2989866

[B14] Harting M. T. Jimenez F. Adams S. D. Mercer D. W. Cox C. S. Jr. (2008). Acute, regional inflammatory response after traumatic brain injury: implications for cellular therapy. Surgery 144, 803–813 10.1016/j.surg.2008.05.017 19081024 PMC3774544

[B15] Israelsson C. Wang Y. Kylberg A. Pick C. G. Hoffer B. J. Ebendal T. (2009). Closed head injury in a mouse model results in molecular changes indicating inflammatory responses. J. Neurotrauma 26, 1307–1314 10.1089/neu.2008.0676 19317611 PMC2989856

[B16] Korfias S. Papadimitriou A. Stranjalis G. Bakoula C. Daskalakis G. Antsaklis A. (2009). Serum biochemical markers of brain injury. Mini Rev. Med. Chem. 9, 227–234 10.2174/138955709787315994 19200027

[B17] Kubal W. S. (2012). Updated imaging of traumatic brain injury. Radiol. Clin. North Am. 50, 15–41 10.1016/j.rcl.2011.08.010 22099485

[B18] Lighthall J. W. (1988). Controlled cortical impact: a new experimental brain injury model. J. Neurotrauma 5, 1–15 10.1089/neu.1988.5.1 3193461

[B19] Loane D. J. Byrnes K. R. (2010). Role of microglia in neurotrauma. Neurotherapeutics 7, 366–377 10.1016/j.nurt.2010.07.002 20880501 PMC2948548

[B20] Medzhitov R. (2008). Origin and physiological roles of inflammation. Nature 454, 428–435 10.1038/nature07201 18650913

[B21] Natale J. E. Ahmed F. Cernak I. Stoica B. Faden A. I. (2003). Gene expression profile changes are commonly modulated across models and species after traumatic brain injury. J. Neurotrauma 20, 907–927 10.1089/089771503770195777 14588109

[B22] Neher M. D. Weckbach S. Flierl M. A. Huber-Lang M. S. Stahel P. F. (2011). Molecular mechanisms of inflammation and tissue injury after major trauma-is complement the “bad guy”? J. Biomed. Sci. 18, 90 10.1186/1423-0127-18-90 22129197 PMC3247859

[B23] Rhodes J. K. Sharkey J. Andrews P. J. (2009). The temporal expression, cellular localization, and inhibition of the chemokines MIP-2 and MCP-1 after traumatic brain injury in the rat. J. Neurotrauma 26, 507–525 10.1089/neu.2008.0686 19210118

[B24] Semple B. D. Bye N. Ziebell J. M. Morganti-Kossmann M. C. (2010). Deficiency of the chemokine receptor CXCR2 attenuates neutrophil infiltration and cortical damage following closed head injury. Neurobiol. Dis. 40, 394–403 10.1016/j.nbd.2010.06.015 20621186

[B25] Sibson N. R. Anthony D. C. Van Kasteren S. Dickens A. Perez-Balderas F. McAteer M. A. (2011). Molecular MRI approaches to the detection of CNS inflammation. Methods Mol. Biol. 711, 379–396 10.1007/978-1-61737-992-5_19 21279613

[B26] Stoll G. Bendszus M. (2009). Imaging of inflammation in the peripheral and central nervous system by magnetic resonance imaging. Neuroscience 158, 1151–1160 10.1016/j.neuroscience.2008.06.045 18651996

[B27] Subramanian A. Tamayo P. Mootha V. K. Mukherjee S. Ebert B. L. Gillette M. A. (2005). Gene set enrichment analysis: a knowledge-based approach for interpreting genome-wide expression profiles. Proc. Natl. Acad. Sci. U.S.A. 102, 15545–15550 10.1073/pnas.0408351102 16199517 PMC1239896

[B28] Svetlov S. I. Larner S. F. Kirk D. R. Atkinson J. Hayes R. L. Wang K. K. (2009). Biomarkers of blast-induced neurotrauma: profiling molecular and cellular mechanisms of blast brain injury. J. Neurotrauma 26, 913–921 10.1089/neu.2008.0609 19422293 PMC6469534

[B29] Venkatesan C. Chrzaszcz M. Choi N. Wainwright M. S. (2010). Chronic upregulation of activated microglia immunoreactive for galectin-3/Mac-2 and nerve growth factor following diffuse axonal injury. J. Neuroinflammation 7, 32 10.1186/1742-2094-7-32 20507613 PMC2891720

[B30] Vitali R. Clarke S. (2004). Improved rotorod performance and hyperactivity in mice deficient in a protein repair methyltransferase. Behav. Brain Res. 153, 129–141 10.1016/j.bbr.2003.11.007 15219714

[B31] Whitney N. P. Eidem T. M. Peng H. Huang Y. Zheng J. C. (2009). Inflammation mediates varying effects in neurogenesis: relevance to the pathogenesis of brain injury and neurodegenerative disorders. J. Neurochem. 108, 1343–1359 10.1111/j.1471-4159.2009.05886.x 19154336 PMC2707502

[B32] Wunder A. Klohs J. Dirnagl U. (2009). Non-invasive visualization of CNS inflammation with nuclear and optical imaging. Neuroscience 158, 1161–1173 10.1016/j.neuroscience.2008.10.005 18983900

